# Peripheral tissue hypoperfusion predicts post intubation hemodynamic instability

**DOI:** 10.1186/s13613-022-01043-3

**Published:** 2022-07-18

**Authors:** Vincent Dubée, Geoffroy Hariri, Jérémie Joffre, Julien Hagry, Lisa Raia, Vincent Bonny, Paul Gabarre, Sebastien Ehrminger, Naike Bigé, Jean-Luc Baudel, Bertrand Guidet, Eric Maury, Guillaume Dumas, Hafid Ait-Oufella

**Affiliations:** 1 Service de Maladies Infectieuses et Tropicales, CHU Angers, Angers, France; 2 Assistance Publique–Hôpitaux de Paris (AP-HP), Hôpital Saint-Antoine, Service de réanimation médicale, 184 rue du Faubourg Saint-Antoine, 75571 Paris Cedex 12, France; 3 Sorbonne Université, Paris, France; 4 Inserm U1136, 75012 Paris, France; 5 Inserm U970, Centre de Recherche Cardiovasculaire de Paris (PARCC), Paris, France

**Keywords:** Intubation, Hemodynamic, Mottling, Tissue perfusion, Outcome

## Abstract

**Background:**

Tracheal intubation and invasive mechanical ventilation initiation is a procedure at high risk for arterial hypotension in intensive care unit. However, little is known about the relationship between pre-existing peripheral microvascular alteration and post-intubation hemodynamic instability (PIHI).

**Methods:**

Prospective observational monocenter study conducted in an 18-bed medical ICU. Consecutive patients requiring tracheal intubation were eligible for the study. Global hemodynamic parameters (blood pressure, heart rate, cardiac function) and tissue perfusion parameters (arterial lactate, mottling score, capillary refill time [CRT], toe-to-room gradient temperature) were recorded before, 5 min and 2 h after tracheal intubation (TI). Post intubation hemodynamic instability (PIHI) was defined as any hemodynamic event requiring therapeutic intervention.

**Results:**

During 1 year, 120 patients were included, mainly male (59%) with a median age of 68 [57–77]. The median SOFA score and SAPS II were 6 [4–9] and 47 [37–63], respectively. The main indications for tracheal intubation were hypoxemia (51%), hypercapnia (13%), and coma (29%). In addition, 48% of patients had sepsis and 16% septic shock. Fifty-one (42%) patients develop PIHI. Univariate analysis identified several baseline factors associated with PIHI, including norepinephrine prior to TI, sepsis, tachycardia, fever, higher SOFA and high SAPSII score, mottling score ≥ 3, high lactate level and prolonged knee CRT. By contrast, mean arterial pressure, baseline cardiac index, and ejection fraction were not different between PIHI and No-PIHI groups. After adjustment on potential confounders, the mottling score was associated with a higher risk for PIHI (adjusted OR: 1.84 [1.21–2.82] per 1 point increased; *p* = 0.005). Among both global haemodynamics and tissue perfusion parameters, baseline mottling score was the best predictor of PIHI (AUC: 0.72 (CI 95% [0.62–0.81]).

**Conclusions:**

In non-selected critically ill patients requiring invasive mechanical ventilation, tissue hypoperfusion parameters, especially the mottling score, could be helpful to predict PIHI.

**Supplementary Information:**

The online version contains supplementary material available at 10.1186/s13613-022-01043-3.

## Introduction

Mechanical ventilation is a central support organ therapy in critically ill patients helpful in case of coma and acute respiratory failure. However, tracheal intubation as well as invasive mechanical ventilation initiation, can induce per se post-intubation hemodynamic instability (PIHI) related to several mechanism including vascular tone alteration related to sedative drugs and impaired venous return consecutive to increased intra-thoracic pressure [1]. The engagement of the sympathetic–renin–angiotensin axis limits the deleterious circulatory impact of MV initiation, promoting vasoconstriction and tachycardia [2]. However, such compensatory mechanisms are altered by general anesthesia, especially in critically ill patients suffering from hypovolemia, hypercapnia or sepsis-related microvascular dysfunction. Arterial hypotension following emergency tracheal intubation has been reported in 10–50% of ICU patients [3–5]. Given that PIHI occurrence worsens the prognosis of critically ill patients [6, 7], the identification of high-risk patients is a major challenge. Several risk factors for PIHI have been reported, including age, high shock index, pH < 7.20, intubation for acute respiratory failure, non-depolarizing agents used for the neuromuscular blockade, and propofol administration [8, 9]. However, the predictive value of tissue perfusion parameters on PIHI has never been evaluated. During the last decade, several bedside “easy to use” tools have been developed to analyze peripheral tissue perfusion including skin mottling, capillary refill time, and toe-to-room temperature gradient [10]. These tissue perfusion parameters are related to organ failure severity and have emerged as strong predictors of unfavorable outcomes in ICU [11–13]. Our group recently found that peripheral tissue hypoperfusion markers predic hemodynamic instability following hemodialysis initiation [14].

In this prospective observational study, we aimed to assess the predictive value of tissue perfusion parameters on PIHI occurrence.

## Patients and methods

### Study subjects and intubation procedure

The study was conducted in an 18-bed medical intensive care unit at a tertiary university hospital. During 1 year, patients requiring intubation were eligible for the study, regardless of the indication for mechanical ventilation. Patients were not included during the night and the weekend, because protocol parameters were recorded by a physician who was not in charge of the patient. The operating physician chose the modalities of anesthesia induction in accordance with the local standard procedure. Briefly, patients received intravenously etomidate (0.3 mg/kg) followed by suxamethonium chloride (1 mg/kg). When etomidate was not used, patients received either propofol or ketamine (2 mg/kg). Curarization in patients with contraindication to suxamethonium was obtained with 1.2 mg/kg rocuronium. Oral tracheal intubation was performed using either a curved blade laryngoscope or a McGrath videolaryngoscope. After tracheal intubation, sedation was maintained using combined sufentanil, midazolam and propofol, according to local standard procedure, with a targeted score of − 2 on the Richmond Agitation–Sedation Scale.

### Data collection and definition of hemodynamic instability

Data were recorded by a physician not in charge of patient care. Data collection included general demographic characteristics and comorbidities, SOFA score on the day of endotracheal intubation, SAPS II score, indication for tracheal intubation, and presence of sepsis. Respiratory and hemodynamic parameters were collected during the hour preceding TI, 5 min and 2 h after tracheal intubation. The difficulty of tracheal intubation was assessed using the Intubation Difficulty Scale [15]. The cardiac index was calculated [16] using transthoracic echocardiography. Mottling score, index and knee capillary refill time (CRT) and toe-to-room gradient temperature were serially measured as previously described [11, 17, 18]. In patients with dark skin, mottling score and knee capillary refill time were not evaluated (*n* = 11 in the study). Arterial blood gas and arterial lactate level were measured routinely using point-of-care testing on a GEM Premier 4000 analyzer. Vasopressor dosage and volume of intravenous fluid administered during the 2 h following intubation were recorded. Hemodynamic instability requiring intervention (PIHI) was defined as any hemodynamic worsening requiring unplanned intravenous fluid resuscitation (≥ 500 ml) or the introduction of vasoactive drugs or an increase in vasoactive drug dose by at least 0.1 µg/kg/min [19, 20]. To limit confounding factors for hemodynamic variations, we only included patients hemodynamically stable before intubation, defined as no increase or introduction of vasoactive support in the hour before intubation.

The ethical committee of the French Intensive Care Society (FICS) approved the protocol (CE SRLF 20-66).

### Statistical analysis

The primary outcome was PIHI as a binary variable. Continuous variables are described as median and interquartile range (IQR) and compared using Wilcoxon’s rank-sum test or Kruskal Wallis; categorical variables are summarized by counts (percents) and compared using exact Fisher test. To evaluate the relationship between microcirculatory parameters and outcome, we used multivariable logistic regression. To avoid overfitting and collinearity, we ran separated models for each parameter. Confounders entered in the models have been selected a priori: sepsis, MAP, drugs used for TI procedure. Log-linearity assumption was checked and variables were tested for collinearity before inclusion in the multivariate model. Goodness of fit was evaluated using Le Cessie–van Houwelingen’s method and discrimination with AUC statistic.

Area under ROC curves (AUROC) was computed using the trapezoidal rule, confidence intervals were determined by the bootstrap technique, and comparison was made as described in DeLong.

The measures of associations are presented with odds ratios and 95% confidence intervals. All tests were two-sided and *p* values lower than 5% were considered to indicate significant associations. Analyses were performed using R statistical platform, version 3.0.2 (https://cran.r-project.org/).

## Results

### Characteristics of included patients

During the study period, 120 patients were included; 41% were female (*n* = 49) with a median age of 68 [57–77] years. Their baseline characteristics are presented in Table 1. The main indications for tracheal intubation were acute respiratory distress with hypoxemia (*n* = 61, 51%) or hypercapnia (*n* = 16, 13%) and coma from non-respiratory cause (*n* = 35, 29%). Most of the patients had sepsis without (*n* = 58, 48%) or with shock (*n* = 18, 16%). Median SOFA score on the day of trachea intubation was 6 [4–9] and SAPS II score was 47 [37–63]. Overall, median score on the Intubation Difficulty Scale was 1 [0–3]. Most of the patients had preserved left ventricular ejection function without right heart failure. Desaturation with a nadir < 90% occurred during the procedure in 36 patients (30%) without any difference between patients with PIHI and patients without (31 vs. 30%). One cardiac arrest occurred following TI for a septic shock patient with severe hypoxia.

**Table 1:** Baseline characteristics of patients included in the study

Patients’ characteristics	Value
Median age, years	68 [57–77]
Male gender	71 (59)
Median SAPS II	47 [37–63]
Median SOFA score	6 [4–9]
Indication for trachea intubation	
Hypoxemia	61 (51)
Hypercapnia	16 (13)
Non-hypercapnic coma	35 (29)
Infection	
No infection	62 (52)
Sepsis	58 (48)
Septic shock	18 (16)
Comorbidities	
Cancer	33 (28)
Liver cirrhosis	4 (3)
Diabetes mellitus	28 (24)
Chronic kidney disease	16 (13)
Cardiovascular disease	24 (20)
Left ventricular ejection fraction before TI	
> 50%	87 (76)
30–50%	18 (16)
< 30%	10 (9)
Right to left ventricular diameter ratio before TI	
< 0.6	77 (66)
0.6–0.9	32 (28)
> 0.9	7 (6)
Presence of paradoxical septum before TI	7 (6)

Data are No. (%) of patients for discontinuous variables, and median [interquartile range] for continuous variables

Data are No. (%) for discontinuous variables, and median [interquartile range] for continuous variables

### Predictors of post intubation hemodynamic instability

#### Baseline characteristics

Fifty-one patients (42%) met the criteria defining PIHI (Table 2) with different interventions: fluid infusion (*N* = 25), norepinephrine introduction (*N* = 6) or dose increase (*N* = 20) (Additional file 1). At baseline, sepsis was more frequently observed in PIHI group (18 vs. 40%; *p* < 0.0001) as well as septic shock (4 vs. 14%; *p* = 0.0004). When compared to No-PIHI group, PIHI patients are characterized by higher central body temperature (37.1 [36.7–37.6] vs. 38.1 [37.4–39.2] °C; *p* = 0.0001), higher SOFA score (5 [4–7] vs. 9 [6–13], *p* < 0.0001) and higher SAPS II score (41 [29–53] vs. 57 [45–69], *p* < 0.0001). There was no significant difference in the Intubation Difficulty Scale between the two groups (0.5 [0; 2.0] vs. 0 [0; 2.7]; *p* = 0.78). Prior to intubation, during resuscitation, patients with PIHI had more often fluid expansion than patients without PIHI (61% vs. 30%; *p* = 0.002). During TI procedure, induction drugs were etomidate (88%) and suxamethonium chloride (55%) or rocuronium (27%) for curarization (Additional file 2).

**Table 2:** Baseline parameters of patients who developed post-intubation hemodynamic instability (PIHI) or not (No PIHI)

Parameters at baseline	No PIHI (*n* = 69)	PIHI (n = 51)	*p* value
Age (years)	70 [58–77]	68 [57–77]	0.76
Male gender, No. (%)	36 (52)	35 (69)	0.10
SAPII score	41 [29–53]	57 [45–69]	< 0.0001
SOFA score	5 [4–7]	9 [6–13]	< 0.0001
Central temperature (°C)	37.1 [36.7–37.6]	38.1 [37.4–39.2]	0.0001
Infection status			
Sepsis, No. (%)	18 (26)	40 (78)	< 0.0001
Septic shock, No. (%)	4 (6)	14 (33)	0.0004
Tissue perfusion markers			
Arterial lactate level (mmol/L)	1.3 [0.9–1.8]	2.3 [1.1–3.5]	0.004
Toe-to-room temperature gradient (°C)	4.4 [1.3–7.7]	2.6 [0.2–7.5]	0.16
Index capillary refill time (s)	1.5 [1–2.1]	1.7 [1.1–2.9]	0.17
Knee capillary refill time (s)	2 [1.3–3.0]	2.9[1.9–5.3]	0.002
Mottling score			
0–1	56 (86)	24 (55)	
[2–3]	7 (11)	11 (25)	
[4–5]	2 (3)	9 (20)	0.0007
Blood gas parameters			
pH	7.42 [7.3–7.46]	7.35 [7.28–7.45]	0.22
P_a_O_2_ (mmHg)	88 [63–161]	89 [69–188]	0.69
P_a_CO_2_ (mmHg)	36 [32–51]	35 [27–40]	0.055
Indication for TI			
Hypoxemia, No. (%)	32 (46)	29 (57)	0.34
Hypercapnia, No. (%)	10 (14)	6 (12)	0.87
Coma from other cause, No. (%)	23 (33)	12 (24)	0.33
Hemodynamic and cardiac function parameters			
Norepinephrine prior to intubation, No. (%)	5 (7)	20 (39)	< 0.0001
Mean arterial blood pressure (mmHg)	87 [78–94]	81 [71–93]	0.14
Cardiac index (L/min/m^2^)	3.2 [2.2–4]	2.8 [2.1–3.7]	0.358
Heart rate (/min)	94 [82–108]	112 [95–130]	0.001
Left ventricular ejection fraction ≤ 30%, No. (%)	5 (7)	5 (11)	0.70
Left to right ventricular diameter ratio ≥ 0.6, No. (%)	24 (35)	15 (32)	0.92

Parameters including baseline characteristics, global hemodynamic and tissue perfusion marker in patients with and without PIHI. Data are No. (%) of patients for discontinuous variables, and median [interquartile range] for continuous variables

#### Global hemodynamic parameters

When compared to No-PIHI group, patients with PIHI had a higher baseline heart rate (94 [82–108] vs. 112 [95–130] bpm; *p* < 0.0001) and higher norepinephrine infusion dose. Cardiac index and baseline both right and left ventricle echocardiographic parameters were not different between groups (Table 2).

#### Peripheral perfusion parameters

Baseline peripheral tissue perfusion parameters were statistically different between No-PIHI and PIHI patients. Patients who developed PIHI had higher arterial lactate level (1.3 [0.9–1.8] vs. 2.3 [1.1–3.5] mmol/L, *p* = 0.004), higher mottling score and prolonged knee capillary refill time (2.0 [1.3–3.0] vs. 2.9 [1.9; 5.3] s, *p* = 0.002) (Table 2).

**Fig. 1:**
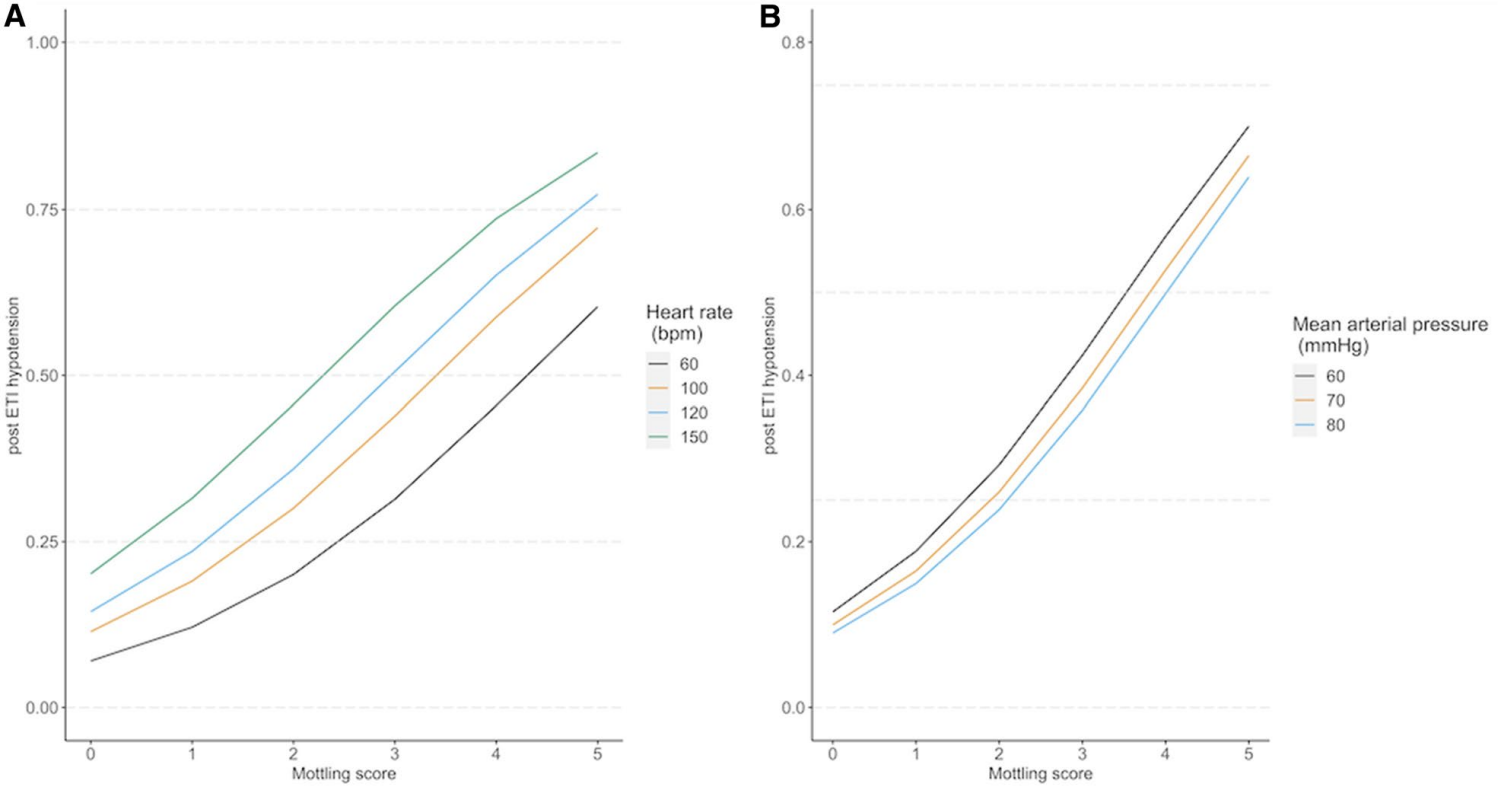
Adjusted probability of PIHI by mottling score as a function of heart rate (**a**) and mean arterial pressure level (**b**)

After adjustment on sepsis, MAP before intubation, and type of medication used during induction, mottling score remains associated with a higher risk of PIHI (adjusted OR: 1.84 [1.21–2.82] per 1 point of mottling score increased; *p* = 0.005) (Table 3). The probability of PIHI according to mottling score and various thresholds of baseline MAP and heart rate is depicted in Fig. 1. In a post-hoc analysis, we re-ran our model with SOFA/SAPS II/vasopressor use instead of MAP and the mottling score remained a strong predictor of PIHI whatever the variable used (Additional file 3).

**Table 3:** Adjusted analysis of variables on the risk of PIHI

Parameters	OR	95CI low	95CI high	*p*
Mottling score (per point)	1.84	1.21	2.82	0.005
Knee CRT	1.23	0.98	1.55	0.075
Index CRT	1.30	0.96	1.75	0.088
Toe to room temp. gradient	0.97	0.86	1.08	0.554
Cardiac output	0.90	0.73	1.11	0.326
Heart rate (per 50 bpm increase)	1.99	0.73	5.42	0.177

Logistic regression analysis. To avoid the risk of over-fitting and collinearity, separate models were used for each variable. The adjustment factors were defined in advance: sepsis, MAP before intubation, type of drug used at induction. The table shows the adjusted ORs

Interestingly, the mottling score remained a strong predictor of PIHI whether patients were receiving vasopressors or not (*p* value for interaction: 0.65, Additional file 4).

**Fig. 2:**
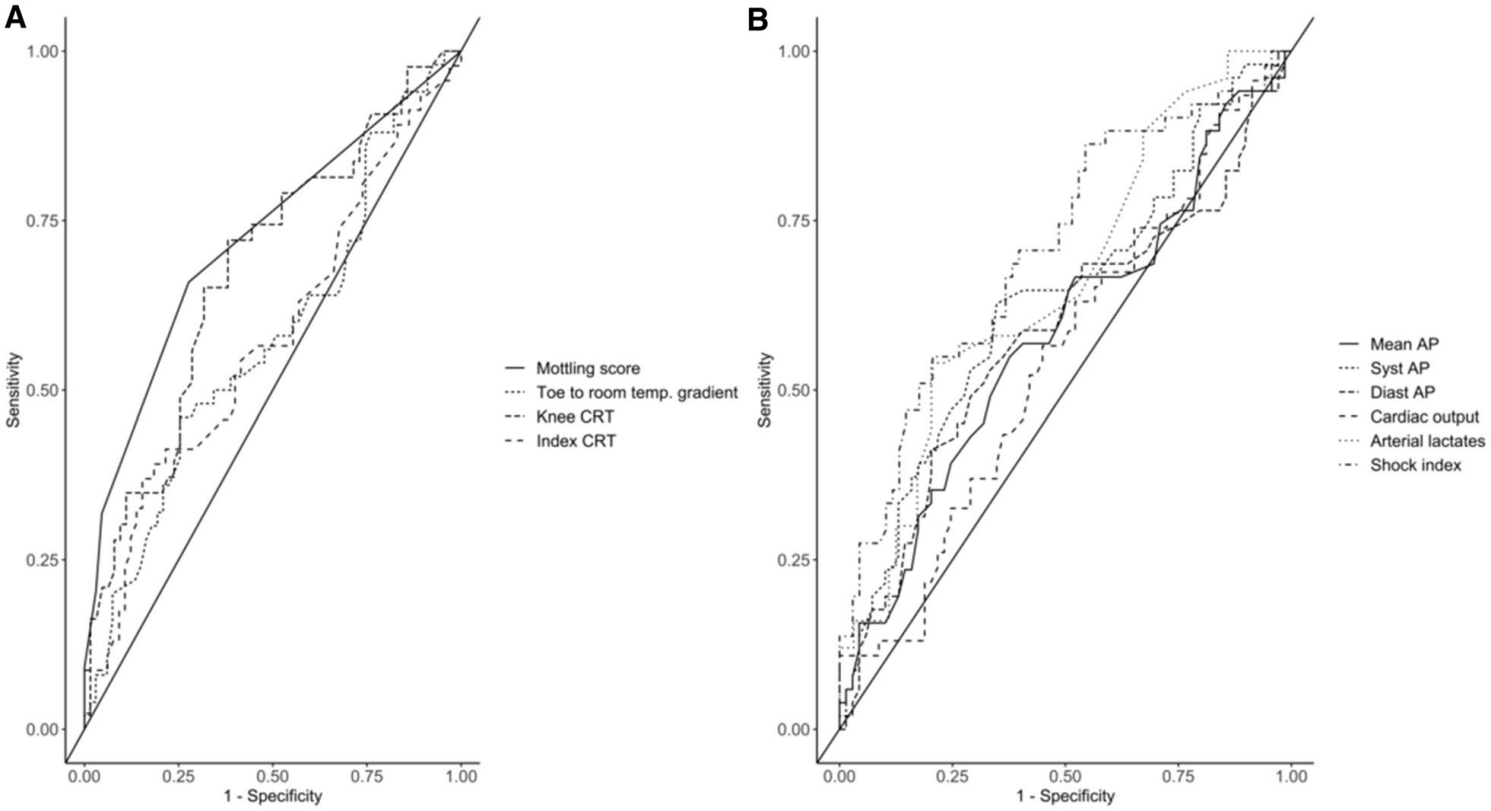
Evaluation of the performance of different skin perfusion (**a**) and hemodynamics’ parameters (**b**) on the risk of post-ETI hypotension

We then compared the ability of selected tissue perfusion and global hemodynamics’ parameters to predict PIHI. As shown in Fig. 2, mottling score before induction was the best predictor of PIHI with an AUC of 0.72 (CI 95% [0.62–0.81]), similar than shock index 0.70 (CI 95% [0.61–0.80]), higher than knee CRT 0.67 (CI 95% [0.57–0.78]), mean arterial pressure 0.58 (CI 95% [0.47–0.68]) or cardiac output 0.54 (CI 95% [0.43–0.65]).

The best threshold of mottling score to predict PIHI was 1, which yielded a specificity of 72.3% CI95% [63.1–81.5]) and sensitivity of 65.9% [54.5–77.3]. The negative predictive value was 75.8% and the positive predictive value was 62%. Performances of other cut-point values of the score are reported in Additional file 5.

Of note, the combination of both mottling score and baseline MAP or Toe-to-room gradient significantly increased the ability to predict PIHI, suggesting that using these tools together could be of interest (Additional file 6).

## Discussion

In this prospective observational study, we found that half of critically ill patients intubated in ICU develop PIHI. We found that disease severity parameters were predictive of PIHI as well as parameters of peripheral tissue hypoperfusion, especially the mottling score.

In a large cohort of ICU patients (*N* = 885), Perbet et al. previously identified independent factors of PIHI, such as high SAPSII score, advanced age, acute respiratory failure as a reason for intubation, first intubation in the ICU, preoxygenation method with noninvasive ventilation and severe hypoxemia [3]. In the emergency department, other risk factors of PIHI have been reported, including chronic obstructive pulmonary disease, sepsis, low body weight and systolic blood pressure lower than 140 mmHg [21]. The shock index, defined as the heart rate divided by the systolic blood pressure [22], is an integrative global hemodynamic tool that has been widely described as a predictor of PIHI in different studies [23, 24].Our study confirmed that SAPS II score, sepsis, need of vasopressors or tachycardia are predictors of PIHI.

In addition, aside from global hemodynamics parameters, we found that peripheral tissue perfusion tools such as mottling score, knee capillary refill are predictive factors for PIHI, with mottling score being the most accurate predictor. Skin mottling has been identified as an independent predictive factor for death in septic shock patients [11, 25]. Mottling that generally develops around the knee area, reflects local decreased microvascular blood flow [26]. In sepsis patients, mottling score correlates with lactate level, urinary output and SOFA suggesting that analysis of skin analysis may be a reliable indicator of global organ perfusion [10]. Using a system combining laser Doppler and iontophoresis [27], our group has shown that endothelial-dependent microvascular reactivity is impaired in the skin knee area suggesting that mottling reflects endothelial dysfunction [28]. The endothelium plays a key role in vasomotor tone and blood pressure regulation in response to acute injury including hypovolemia [29]. Intermittent hemodialysis is a common condition on ICU which could be responsible for acute hypovolemia due to partial redistribution of blood volume from the intravascular compartment to the extracorporeal circuit. In this context, we have previously shown that mottling score is a strong predictor of arterial hypotension following intermittent hemodialysis initiation [14]. Similarly, tracheal intubation and mechanical ventilation initiation also induce acute hypovolemia in ICU due to sedative drug-induced vasoplegia and decreased venous return due to intra-thoracic venous pressure. In this study, we also identified that mottling score predicts PIHI after adjustment to global hemodynamic parameters, such as blood pressure. Such observation suggests that analysis of peripheral perfusion should be helpful before TI to evaluate the risk for PIHI.

An intubation management protocol has been developed to limit the occurrence of life-threatening complications after intubation [30]. This global strategy including pre-intubation fluid infusion provided convincing results. However, two recent randomized controlled trials challenged the benefit of systematic fluid infusion before intubation to prevent PIHI in a non-selected population of critically ill patients [31, 32]. A combination of tissue perfusion parameters and MAP, which increased the prediction of PIHI, may be of interest for tailored intervention and to limit fluid overload, associated with poor outcome in ICU [33]. In addition, the identification of patients at high risk for PIHI may be helpful for the choice of the induction drug. Based on a recent randomized trial showing that ketamine use is associated with reduced cardiovascular collapse after intubation and improved day-7 survival when compared to etomidate, ketamine should be recommended in patients at high risk for PIHI [34]

According to Perbet et al. [3], propofol use was associated with less PIHI, this observation should be taken with caution, whereas etomidate or ketamine are known to be well tolerated [35, 36] and may be the result of the selection of hemodynamically stable patients for propofol induction drugs.

Our study has several limitations. First, itis a monocentric study and results need to be confirmed in a multicenter study including a larger population. However, we analyzed more than one hundred intubations in a non-selected medical ICU population. Protocoled use of sedative drugs was not standardized in our study, but it reflected the actual practice for rapid sequence intubation in ICU, with large use of etomidate, suxamethonium or rocuronium.

Several patients were treated with vasoactive drug before TI but mottling score remains predictive of PIHI whether patient received vasopressor or not. Another issue is the difficulty of analyzing the mottled skin on patients with dark skin. In this case, the toe-to-room temperature gradient could be helpful [10, 18].

## Conclusions

In this prospective study on ICU patients requiring invasive mechanical ventilation, half of the patients required fluid resuscitation or vasoactive drugs in the 2 h following TI. Skin perfusion parameters, especially pre-intubation mottling score were the strongest predictor of hemodynamic worsening.

## Supplementary Information

**Additional file 1.** Hemodynamic and tissue perfusion parameters at different timepoints in patients without (A) or with PIHI (B). Data are expressed as medians [interquartile range] for continuous variables, and No. (%) of patients for categorical variables. ND, not determined. a, Wilcoxon matched paired test; b, comparison of data observed before vs. 5 min after TI; c, comparison of data observed before vs. 2 h after TI; d, comparison of data observed 5 min after TI vs. 2 h after TI;CRT, capillary refill time, MAP, mean arterial pressure.

**Additional file 2.** Induction drugs used for patients who developed post-intubation PIHI or not.

**Additional file 3.** Effect of mottling score (per point increase) on PIHI incidence adjusted on various hemodynamic/severity parameters.*Each separate model is also adjusted on sepsis and induction drugs.

**Additional file 4.** Mottling score effect on post-tracheal intubation hypotension according to vasopressor use before tracheal intubation.

**Additional file 5.** Performances of cut-point values of the mottling score to predict PIHI.

**Additional file 6.** The delta AUROC column shows the increase in discrimination, as measured by the AUROC, due to addition of mottling score to the variable indicated in the first column (logistic regression model using mottling score together with the variable in the first column).
