# Supplementary material for: Peripheral tissue hypoperfusion predicts post intubation hemodynamic instability
Source: Ann Intensive Care. 2022 Jul 18;12:68. doi: 10.1186/s13613-022-01043-3 (PMC9288942; doi:10.1186/s13613-022-01043-3)
Supplement: Supplementary file 1 — Additional file 1. Hemodynamic and tissue perfusion parameters at different timepoints in patients without (A) or with PIHI (B). Data are expressed as medians [interquartile range] for continuous variables, and No. (%) of patients for categorical variables. ND, not determined. a, Wilcoxon matched paired test; b, comparison of data observed before vs. 5 min after TI; c, comparison of data observed before vs. 2 h after TI; d, comparison of data observed 5 min after TI vs. 2 h after TI;CRT, capillary refill time, MAP, mean arterial pressure. [file 13613_2022_1043_MOESM1_ESM.pptx]

## Slide 1
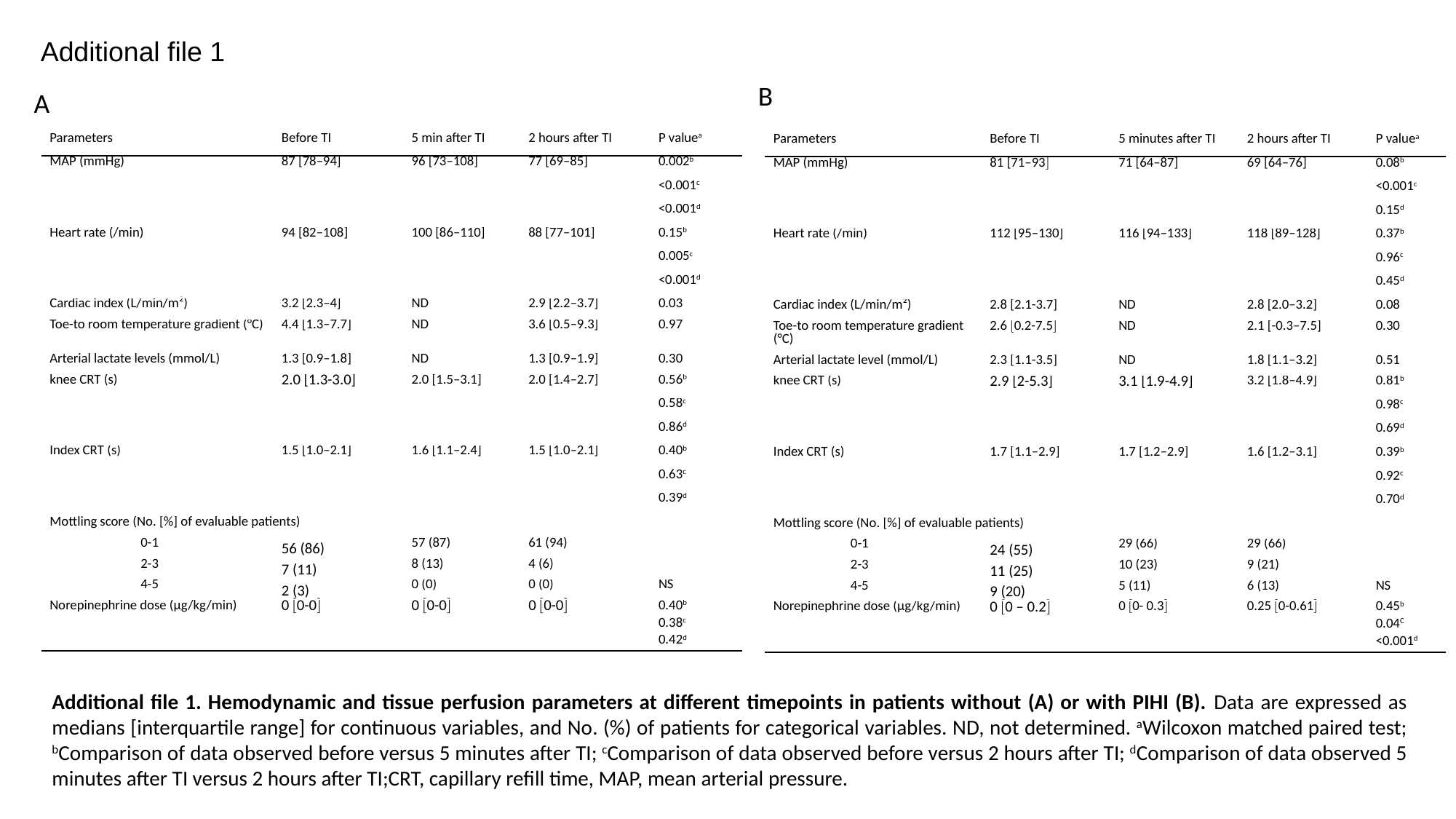

Additional file 1
B
A
| Parameters | | Before TI | 5 min after TI | 2 hours after TI | P valuea |
| --- | --- | --- | --- | --- | --- |
| MAP (mmHg) | | 87 [78–94] | 96 [73–108] | 77 [69–85] | 0.002b |
| | | | | | <0.001c |
| | | | | | <0.001d |
| Heart rate (/min) | | 94 [82–108] | 100 [86–110] | 88 [77–101] | 0.15b |
| | | | | | 0.005c |
| | | | | | <0.001d |
| Cardiac index (L/min/m²) | | 3.2 [2.3–4] | ND | 2.9 [2.2–3.7] | 0.03 |
| Toe-to room temperature gradient (°C) | | 4.4 [1.3–7.7] | ND | 3.6 [0.5–9.3] | 0.97 |
| Arterial lactate levels (mmol/L) | | 1.3 [0.9–1.8] | ND | 1.3 [0.9–1.9] | 0.30 |
| knee CRT (s) | | 2.0 [1.3-3.0] | 2.0 [1.5–3.1] | 2.0 [1.4–2.7] | 0.56b |
| | | | | | 0.58c |
| | | | | | 0.86d |
| Index CRT (s) | | 1.5 [1.0–2.1] | 1.6 [1.1–2.4] | 1.5 [1.0–2.1] | 0.40b |
| | | | | | 0.63c |
| | | | | | 0.39d |
| Mottling score (No. [%] of evaluable patients) | | | | | |
| | 0-1 | 56 (86) | 57 (87) | 61 (94) | |
| | 2-3 | 7 (11) | 8 (13) | 4 (6) | |
| | 4-5 | 2 (3) | 0 (0) | 0 (0) | NS |
| Norepinephrine dose (µg/kg/min) | | 0 0-0 | 0 0-0 | 0 0-0 | 0.40b |
| | | | | | 0.38c |
| | | | | | 0.42d |
| Parameters | | Before TI | 5 minutes after TI | 2 hours after TI | P valuea |
| --- | --- | --- | --- | --- | --- |
| MAP (mmHg) | | 81 [71–93 | 71 [64–87] | 69 [64–76] | 0.08b |
| | | | | | <0.001c |
| | | | | | 0.15d |
| Heart rate (/min) | | 112 [95–130] | 116 [94–133] | 118 [89–128] | 0.37b |
| | | | | | 0.96c |
| | | | | | 0.45d |
| Cardiac index (L/min/m²) | | 2.8 [2.1-3.7] | ND | 2.8 [2.0–3.2] | 0.08 |
| Toe-to room temperature gradient (°C) | | 2.6 0.2-7.5 | ND | 2.1 [-0.3–7.5] | 0.30 |
| Arterial lactate level (mmol/L) | | 2.3 [1.1-3.5] | ND | 1.8 [1.1–3.2] | 0.51 |
| knee CRT (s) | | 2.9 [2-5.3] | 3.1 [1.9-4.9] | 3.2 [1.8–4.9] | 0.81b |
| | | | | | 0.98c |
| | | | | | 0.69d |
| Index CRT (s) | | 1.7 [1.1–2.9] | 1.7 [1.2–2.9] | 1.6 [1.2–3.1] | 0.39b |
| | | | | | 0.92c |
| | | | | | 0.70d |
| Mottling score (No. [%] of evaluable patients) | | | | | |
| | 0-1 | 24 (55) | 29 (66) | 29 (66) | |
| | 2-3 | 11 (25) | 10 (23) | 9 (21) | |
| | 4-5 | 9 (20) | 5 (11) | 6 (13) | NS |
| Norepinephrine dose (µg/kg/min) | | 0 0 – 0.2 | 0 0- 0.3 | 0.25 0-0.61 | 0.45b |
| | | | | | 0.04C |
| | | | | | <0.001d |
Additional file 1. Hemodynamic and tissue perfusion parameters at different timepoints in patients without (A) or with PIHI (B). Data are expressed as medians [interquartile range] for continuous variables, and No. (%) of patients for categorical variables. ND, not determined. aWilcoxon matched paired test; bComparison of data observed before versus 5 minutes after TI; cComparison of data observed before versus 2 hours after TI; dComparison of data observed 5 minutes after TI versus 2 hours after TI;CRT, capillary refill time, MAP, mean arterial pressure.
