# Supplementary material for: Peripheral tissue hypoperfusion predicts post intubation hemodynamic instability
Source: Ann Intensive Care. 2022 Jul 18;12:68. doi: 10.1186/s13613-022-01043-3 (PMC9288942; doi:10.1186/s13613-022-01043-3)
Supplement: Supplementary file 2 — Additional file 2. Induction drugs used for patients who developed post-intubation PIHI or not. [file 13613_2022_1043_MOESM2_ESM.pptx]

## Slide 1
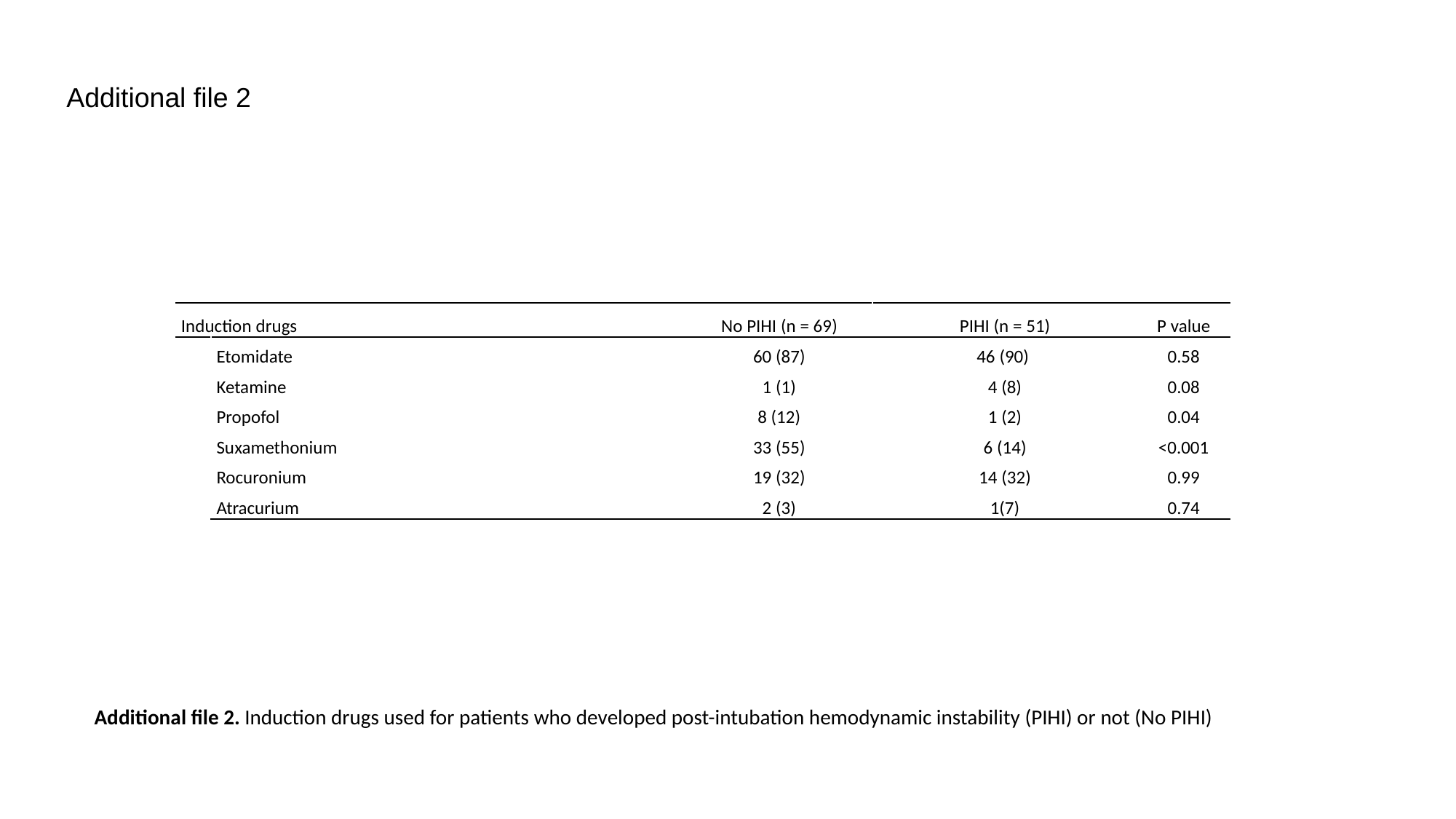

Additional file 2
| | | | | | |
| --- | --- | --- | --- | --- | --- |
| Induction drugs | | | No PIHI (n = 69) | PIHI (n = 51) | P value |
| | Etomidate | Etomidate | 60 (87) | 46 (90) | 0.58 |
| | Ketamine | Ketamine | 1 (1) | 4 (8) | 0.08 |
| | Propofol | Propofol | 8 (12) | 1 (2) | 0.04 |
| | Suxamethonium | Suxamethonium | 33 (55) | 6 (14) | <0.001 |
| | Rocuronium | Rocuronium | 19 (32) | 14 (32) | 0.99 |
| | Atracurium | Atracurium | 2 (3) | 1(7) | 0.74 |
Additional file 2. Induction drugs used for patients who developed post-intubation hemodynamic instability (PIHI) or not (No PIHI)
