# Supplementary material for: Peripheral tissue hypoperfusion predicts post intubation hemodynamic instability
Source: Ann Intensive Care. 2022 Jul 18;12:68. doi: 10.1186/s13613-022-01043-3 (PMC9288942; doi:10.1186/s13613-022-01043-3)
Supplement: Supplementary file 3 — Additional file 3. Effect of mottling score (per point increase) on PIHI incidence adjusted on various hemodynamic/severity parameters.*Each separate model is also adjusted on sepsis and induction drugs. [file 13613_2022_1043_MOESM3_ESM.pptx]

## Slide 1
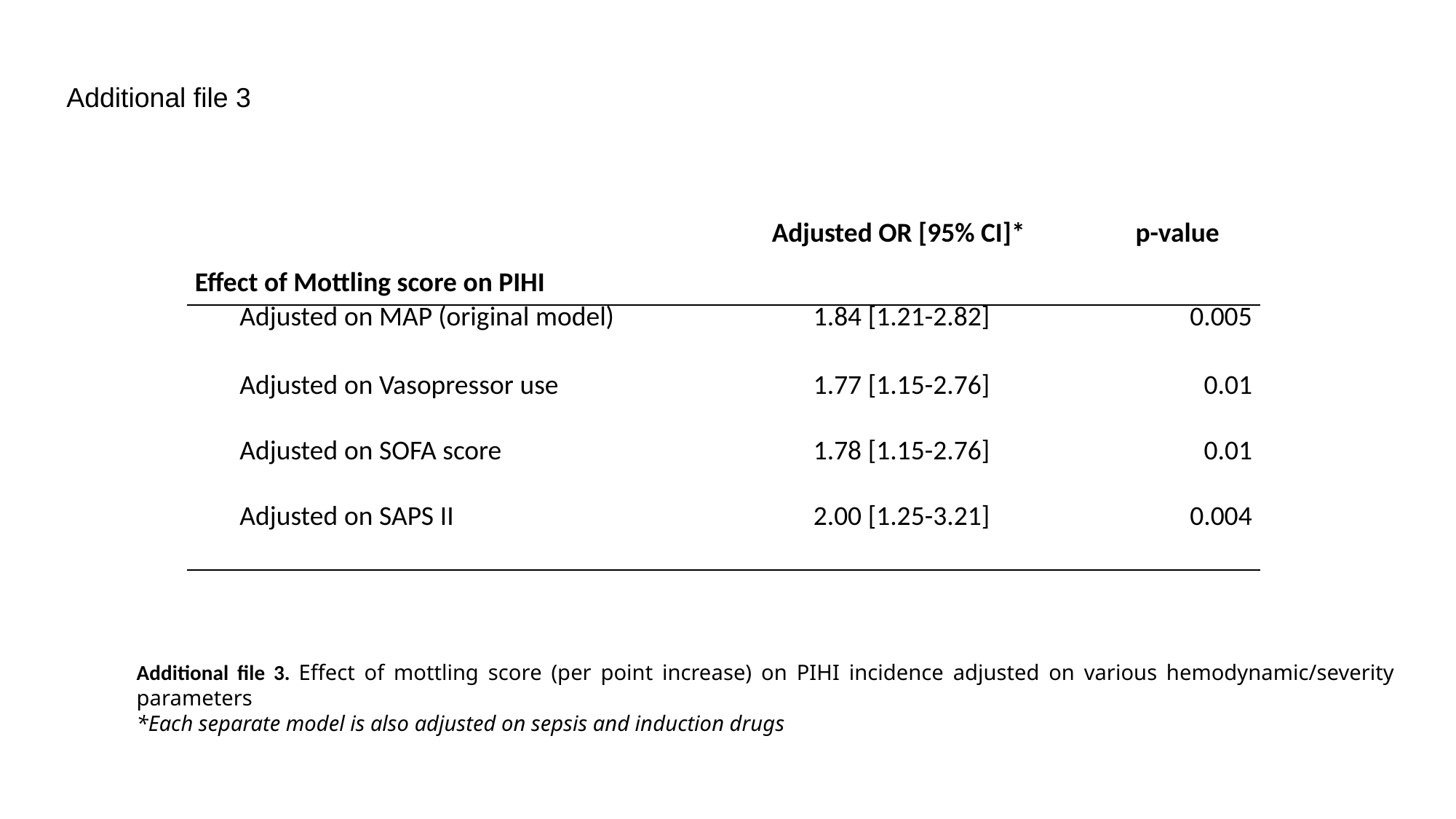

Additional file 3
| | Adjusted OR [95% CI]\* | p-value |
| --- | --- | --- |
| Effect of Mottling score on PIHI | | |
| Adjusted on MAP (original model) | 1.84 [1.21-2.82] | 0.005 |
| Adjusted on Vasopressor use | 1.77 [1.15-2.76] | 0.01 |
| Adjusted on SOFA score | 1.78 [1.15-2.76] | 0.01 |
| Adjusted on SAPS II | 2.00 [1.25-3.21] | 0.004 |
Additional file 3. Effect of mottling score (per point increase) on PIHI incidence adjusted on various hemodynamic/severity parameters
*Each separate model is also adjusted on sepsis and induction drugs
