# Supplementary material for: Peripheral tissue hypoperfusion predicts post intubation hemodynamic instability
Source: Ann Intensive Care. 2022 Jul 18;12:68. doi: 10.1186/s13613-022-01043-3 (PMC9288942; doi:10.1186/s13613-022-01043-3)
Supplement: Supplementary file 4 — Additional file 4. Mottling score effect on post-tracheal intubation hypotension according to vasopressor use before tracheal intubation. [file 13613_2022_1043_MOESM4_ESM.pptx]

## Slide 1
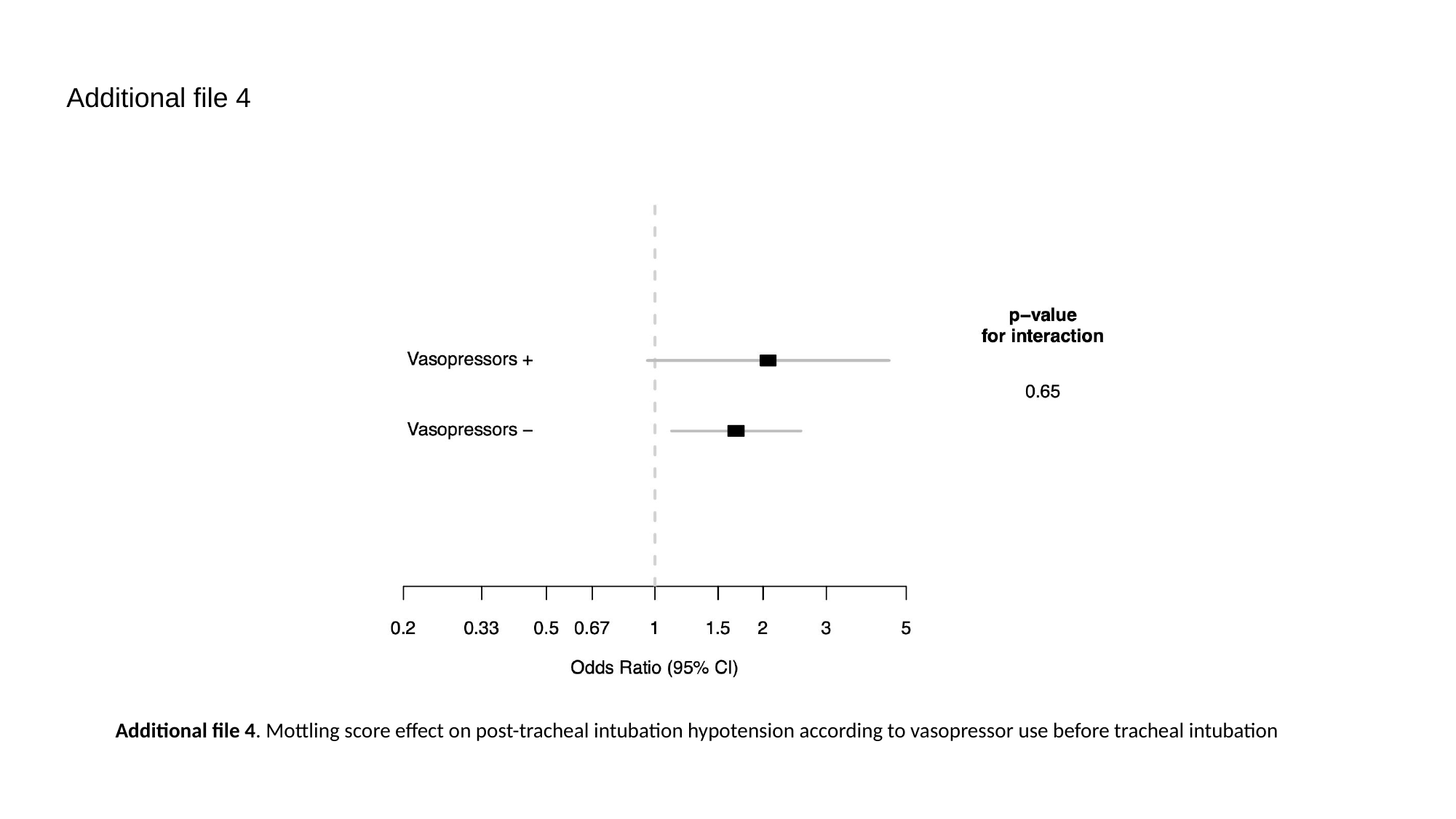

Additional file 4
Additional file 4. Mottling score effect on post-tracheal intubation hypotension according to vasopressor use before tracheal intubation
