# Supplementary material for: Peripheral tissue hypoperfusion predicts post intubation hemodynamic instability
Source: Ann Intensive Care. 2022 Jul 18;12:68. doi: 10.1186/s13613-022-01043-3 (PMC9288942; doi:10.1186/s13613-022-01043-3)
Supplement: Supplementary file 5 — Additional file 5. Performances of cut-point values of the mottling score to predict PIHI. [file 13613_2022_1043_MOESM5_ESM.pptx]

## Slide 1
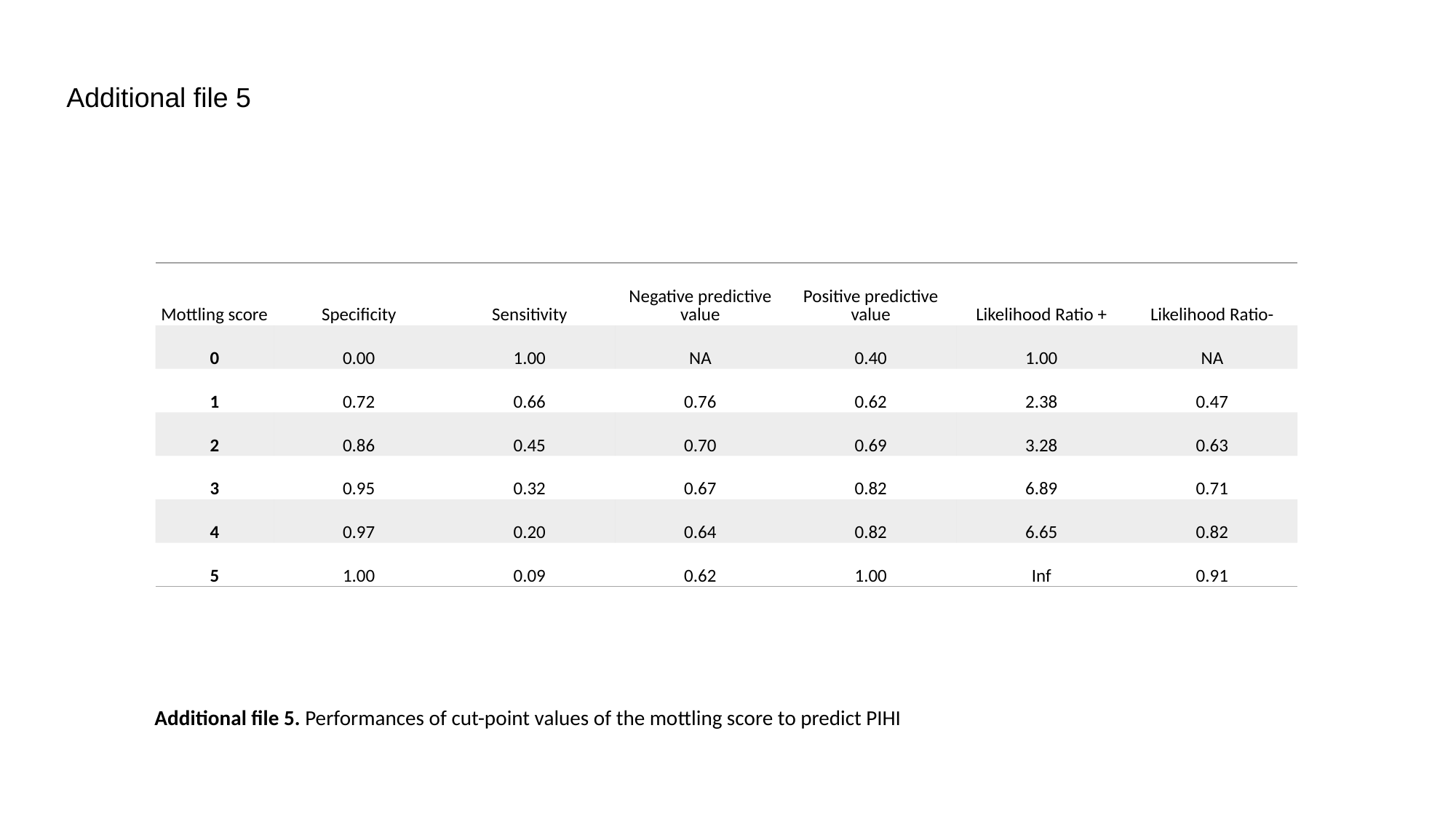

Additional file 5
| Mottling score | Specificity | Sensitivity | Negative predictive value | Positive predictive value | Likelihood Ratio + | Likelihood Ratio- |
| --- | --- | --- | --- | --- | --- | --- |
| 0 | 0.00 | 1.00 | NA | 0.40 | 1.00 | NA |
| 1 | 0.72 | 0.66 | 0.76 | 0.62 | 2.38 | 0.47 |
| 2 | 0.86 | 0.45 | 0.70 | 0.69 | 3.28 | 0.63 |
| 3 | 0.95 | 0.32 | 0.67 | 0.82 | 6.89 | 0.71 |
| 4 | 0.97 | 0.20 | 0.64 | 0.82 | 6.65 | 0.82 |
| 5 | 1.00 | 0.09 | 0.62 | 1.00 | Inf | 0.91 |
Additional file 5. Performances of cut-point values of the mottling score to predict PIHI
