# Supplementary material for: Peripheral tissue hypoperfusion predicts post intubation hemodynamic instability
Source: Ann Intensive Care. 2022 Jul 18;12:68. doi: 10.1186/s13613-022-01043-3 (PMC9288942; doi:10.1186/s13613-022-01043-3)
Supplement: Supplementary file 6 — Additional file 6. The delta AUROC column shows the increase in discrimination, as measured by the AUROC, due to addition of mottling score to the variable indicated in the first column (logistic regression model using mottling score together with the variable in the first column). [file 13613_2022_1043_MOESM6_ESM.pptx]

## Slide 1
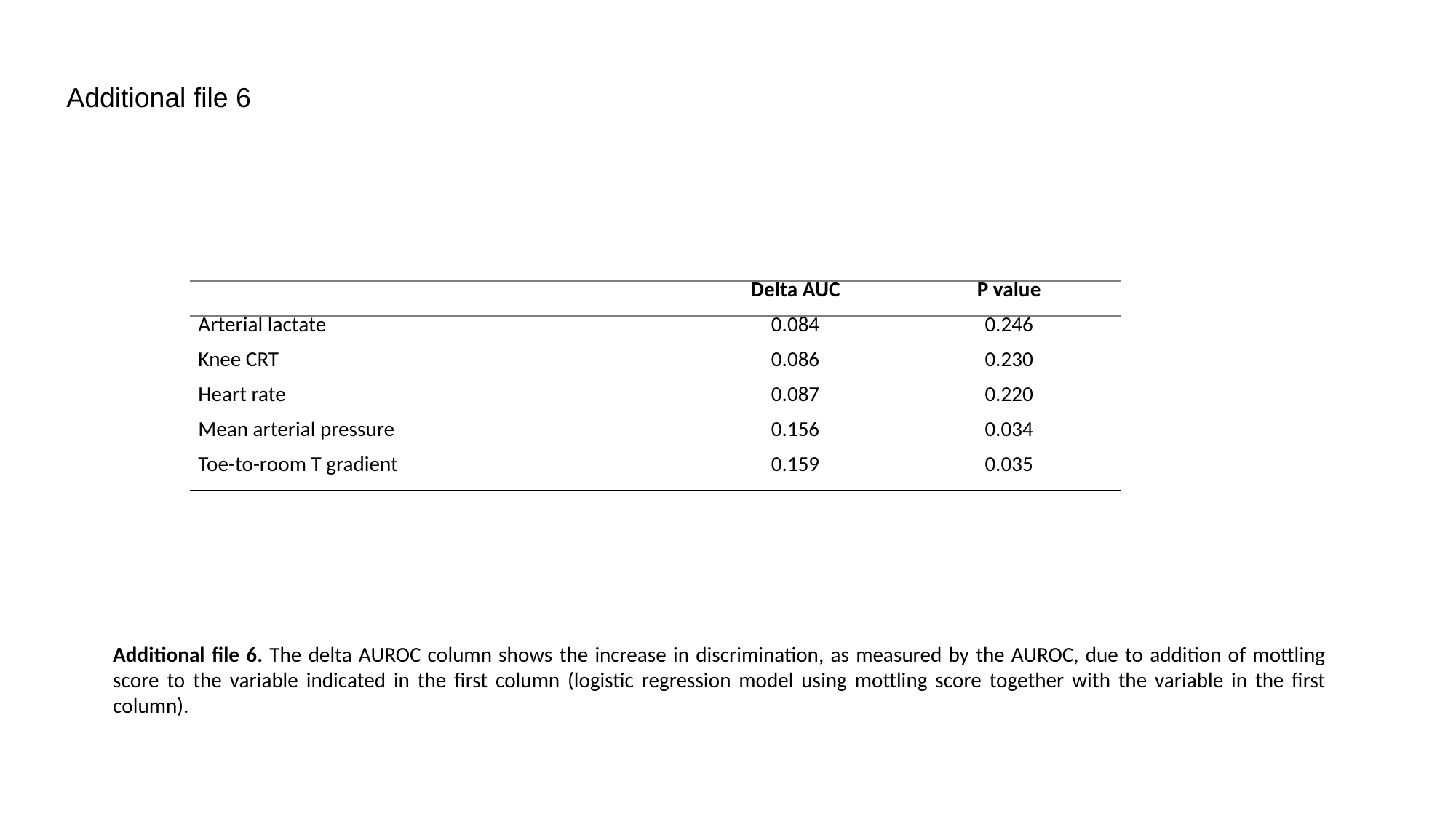

Additional file 6
| | Delta AUC | P value |
| --- | --- | --- |
| Arterial lactate | 0.084 | 0.246 |
| Knee CRT | 0.086 | 0.230 |
| Heart rate | 0.087 | 0.220 |
| Mean arterial pressure | 0.156 | 0.034 |
| Toe-to-room T gradient | 0.159 | 0.035 |
Additional file 6. The delta AUROC column shows the increase in discrimination, as measured by the AUROC, due to addition of mottling score to the variable indicated in the first column (logistic regression model using mottling score together with the variable in the first column).
